# Targeted FT-NIR and SERS Detection of Breast Cancer HER-II Biomarkers in Blood Serum Using PCB-Based Plasmonic Active Nanostructured Thin Film Label-Free Immunosensor Immobilized with Directional GNU-Conjugated Antibody

**DOI:** 10.3390/s24165378

**Published:** 2024-08-20

**Authors:** Mohammad E. Khosroshahi, Yesha Patel, Vithurshan Umashanker

**Affiliations:** 1 Nanobiophotonics & Biomedical Research Laboratory, M.I.S. Electronics Inc., Richmond Hill, ON L4B 1B4, Canada; 2 Institute for Advanced Non-Destructive and Non-Invasive Diagnostic Technologies (IANDIT), University of Toronto, Toronto, ON M5S 3G8, Canada; 3 Department of Mechanical and Industrial Engineering, University of Toronto, Toronto, ON M5S 3G8, Canada; 4 Department of Biochemical Engineering, University of Waterloo, Waterloo, ON N2L 3G1, Canada; 5 Department of Electrical and Computer Engineering, University of Waterloo, Waterloo, ON N2L 3G1, Canada

**Keywords:** SERS, gold nanourchins, breast cancer, human serum, biomarker, directional antibody

## Abstract

This work describes our recent PCB-based plasmonic nanostructured platform patent (US 11,828,747B2) for the detection of biomarkers in breast cancer serum (BCS). A 50 nm thin gold film (TGF) was immersion-coated on PCB (i.e., PCB-TGF) and immobilized covalently with gold nanourchin (GNU) via a 1,6-Hexanedithiol (HDT) linkage to produce a plasmonic activated nanostructured thin film (PANTF) platform. A label-free SERS immunosensor was fabricated by conjugating the platform with monoclonal HER-II antibodies (mAb) in a directional orientation via adipic acid dihydrazide (ADH) to provide higher accessibility to overexpressed HER-II biomarkers (i.e., 2+ (early), 3+ (locally advanced), and positive (meta) in BCS. An enhancement factor (EF) of 0.3 × 10^5^ was achieved for PANTF using Rhodamine (R6G), and the morphology was studied by scanning electron microscopy (SEM) and atomic force microscope (AFM). UV-vis spectroscopy showed the peaks at 222, 231, and 213 nm corresponding to ADH, mAb, and HER-II biomarkers, respectively. The functionalization and conjugation were investigated by Fourier Transform Near Infrared (FT-NIR) where the most dominant overlapped spectra of 2+, 3+, and Pos correspond to OH-combination of carbohydrate, RNH_2_ 1st overtone, and aromatic CH 1st overtone of mAb, respectively. SERS data were filtered using the *filtfilt* filter from *scipy.signals*, baseline corrected using the Improved Asymmetric Least Squares (*isals*) function from the *pybaselines.Whittaker* library. The results showed the common peaks at 867, 1312, 2894, 3026, and 3258 cm^−1^ corresponding to glycine, alanine *ν* (C-N-C) assigned to the symmetric C-N-C stretch mode; tryptophan and α helix; C-H antisymmetric and symmetric stretching; NH_3_^+^ in amino acids; and N-H stretch primary amide, respectively, with the intensity of Pos > 3^+^ > 2^+^. This trend is justifiable considering the stage of each sample. Principal Component Analysis (PCA) and Linear Discrimination Analysis (LDA) were employed for the statistical analysis of data.

## 1. Introduction

Cancer is a multi-stage and complex disease characterized by uncontrolled genomic and chromosomal abnormalities resulting from genetic modification and molecular changes that alter the functionality of the cell and its proliferation. It is considered one of the most significant global public health challenges, a life-threatening and widespread disease causing substantial morbidity and mortality [1,2]. Although efficient therapeutic mediation can be performed at the early stages of the development of cancer, the delay in diagnosing the developed stages makes efficient treatments ineffective. Breast cancer (BC), as the most prevalent neoplasm in women and the second leading cause of global mortality [3], particularly affecting developing countries [3,4], stands out because of its molecular heterogeneity, morphological and clinical characteristics, hence leading to complicated subtypes with distinct clinical outcomes and responses to treatment [5]. HER-II overexpression is one of the earliest events during breast carcinogenesis, and its serum levels are considered a promising real-time marker for the presence or recurrence of tumors and triple-negative (TNBC), which is characterized by the lack of expression of any of the above receptors [6,7].

The current techniques used for the BC diagnosis include palpation, which to a great extent depends on the physician’s skill and experience and is limited by the size and depth of the tumor; biopsy or excision is invasive and time-consuming, and other frequently utilized medical devices including mammography (X-ray), magnetic resonance imaging (MRI), ultrasound (US), computerized tomography (CT), single-photon emission computed tomography (SPECT), and positron emission tomography (PET) each possess unique advantages and limitations. Some primary limitations include inherent invasiveness, utilization of ionizing radiation and radioactive agents such as gadolinium injections, and incompatibility with patients who have pacemakers or breast reconstructions. Moreover, these techniques necessitate costly and specialized facilities for managing radiopharmaceuticals, employ iodine-based compounds, and exhibit constraints in sensitivity and image spatial resolution. Consequently, this increases the risk of both false positive and false negative results, directly impacting the patient’s psychology, confidence, and morale. Indeed, the development of advanced point-of-care (POC) devices for early diagnosis of cancer that meet these requirements represents a critical and challenging medical priority. This effort is motivated by the growing demand to minimize unnecessary biopsies of benign tissues and mitigate associated physical and psychological concerns. These techniques should be non-invasive, reliable, real-time, accurate, easily measurable, cost-effective, and yield rapid and dependable results.

Optical biosensors, as a powerful alternative to conventional analytical methods, have great potential for the direct, real-time, and label-free detection of several chemical and biological substances. The main objective is to produce an electronic signal with an intensity or frequency proportional to the concentration of a specific analyte or group of analytes. For active biosensing, selective binding of the target analyte to a surface-conjugated ligand, i.e., a bioreceptor, is needed. Biosensors are sensitive, selective, portable, and compact, require a minimum amount of specimen, have a low signal-to-noise ratio, and are able to perform remote sensing [8]. The most significant characteristic of immunosensors is the specificity of antigen–antibody reactions, which leads them to become an appealing candidate for the progress of diagnostic tools as POC instruments, which supply complete automation, quick response, cheap, great sensitivity, portability, precision, and accuracy [9,10]. Contrary to immunoassay, label-free immunosensors, or in other words direct-immunosensors, have the capability to detect the chemical or physical alterations stem directly from the immune antibody–antigen compound generation without requiring a label. However, to improve early cancer diagnosis, it is essential to recognize and promptly determine the cancer-specific biomarkers accurately.

A biomarker is a biochemical substance that serves a vital role in cancer diagnosis because of special genomic alterations in cancerous cells with the progress of the disease. It is divided into two broad categories: (a) cellular and (b) humoral. The first type is associated with tumorous cells or tumor antigens, which are expressed via tumors and are used to diagnose cancer. The latter is secreted either by the tumor itself or during the tumor disintegration into the biofluid, including blood and serum specimens of the patients [11]. Blood-based biomarkers have significant importance in non-invasive cancer screening and in reducing the total national economic burden [12]. Analyzing the biomarkers at early stages and low concentrations and characterizing their pathology may be able to provide information about early tumor development to detect cancer such as BC at an early and more treatable stage, enhancing the survival of cancer patients [13,14]. Although the detection of biomarkers is not the primary diagnostic appliance for cancer, they can be utilized as a complementary and complete strategy for supporting the diagnosis.

BCS represents the blood plasma, which consists of the standard contents such as proteins, antibodies, vitamins, minerals, and other materials except the clotting agent, i.e., fibrinogen. The advantages of non-invasive technique include (a) non-destructive and without direct contact with the body, so the invasive biopsy or external body intervention is eliminated; (b) rapid, real-time, inexpensive, and accurate assessment of samples such as biofluid or tissue; (c) in-vitro-assessment increases the safety by being non-contact and avoiding the possible infection; (d) valuable information can be provided on a patient’s health conditions; (e) early diagnosis helps physicians in decision making and plan treatment on time.

The Raman spectroscopy (RS) technique is one of the most effective methods of identifying molecular species and the chemical composition of an analyte via an inelastic Raman scattering referred to as vibrational fingerprinting, which is obtained by recording specific vibrational modes of sampled molecules. Raman frequency shifts during the spectroscopy have been widely used in cancer diagnosis to identify the corresponding biochemicals in healthy and cancerous tissues since the latter has an excess amount of (i.e., concentration) of proteins, carbohydrates, lipids, and amino acids, which play a key role in disease detection [15,16].

However, the intensity of Raman signals is normally weak and needs to be enhanced for better efficiency and usefulness. This is conducted by surface-enhanced Raman scattering (SERS) as a powerful technique to enhance the Raman signal of analyte molecules situated in the near vicinity of plasmonic nanoparticles (PNPs). These PNPs exhibit strong optical absorption and scattering because of localized surface plasmon resonance (LSPR) [16,17]. SERS substrates with specific plasmonic characteristics can enhance the Raman signal intensity of adsorbed molecules by several orders of magnitudes because of the formation of hot spots where LSPR occurs. The geometry and dimension of nanostructures impact the spectral properties because of changes in surface polarization. Among the variety of PNPs, gold NPs of various shapes and sizes exhibit interesting properties such as strong surface absorption and scattering and LSPR, tunable SPR in the Vis-NIR, time-dependent properties because of adjustable nanoparticle size and shape [18,19,20,21], nontoxicity, biocompatibility because of their inert surface, easy synthesis, functionalization and surface conjugation chemistry no photobleaching, and very low oxidation [17,22].

When a PNP is exposed to an external time-varying sinusoidal electric field, polarization occurs because of the displacement of the negative conduction band electron gas with respect to the nucleus positive ions, thus creating a dipole moment. The Coulomb interaction generates a restoring force $F_{r}=-m_{e}\omega_{0}^{2}$ where $m_{e}$ is the mass of the electron and $\omega_{0}$ is the oscillation angular frequency that brings the electrons back to its initial position, and the surface plasmon-SP (i.e., the collective excitation of the free conductive electron gas at the metal-dielectric interface) undergoes a continuous collective oscillation until damping forces bring the system to rest. Plasmon frequency $\omega_{p}$ occurs when the frequency of the incident light equals the frequency of maximum amplitude associated with electron oscillation (i.e., dipolar plasmon)

(1) $$\omega_{p}^{2}=\frac{ne^{2}}{m\varepsilon_{0}}$$

where n is the density of electrons, e is the electron charge (1.6 × 10^−19^ C), and $\varepsilon_{0}$ is the vacuum dielectric constant permittivity (8.85 × 10^−12^ Fm^−1^). It is established that as the size of the NPs increases, SPs become more significant, which results in redshifts of the LSPR wavelength (i.e., the plasmon absorption band). Therefore, the collective oscillation of electrons decreases, leading to wider plasmon bandwidths and lower SERS enhancement [23]. The polarizability of a small spherical NP smaller than the wavelength of the light is

(2) $$\alpha =4\pi \varepsilon_{0}R_{p}^{3}\left(\frac{\varepsilon_{m}(\omega )-\varepsilon_{s}}{\varepsilon_{m}(\omega )+2\varepsilon_{s}}\right)$$

where $R_{p}$ is the radius of NP, $\varepsilon_{m}(\omega )=\varepsilon_{r}-i\varepsilon_{i}$ and $\varepsilon_{s}$ are the complex dielectric permittivity of metal and the surrounding medium, respectively.

(3) $$\varepsilon_{r}=1-\frac{\omega_{p}^{2}}{\omega^{2}+\gamma^{2}}$$

is the real part that determines the degree to which the metal polarizes in response to an applied external electricity and

(4) $$\varepsilon_{i}=\frac{\omega_{p}^{2}\gamma }{\omega (\omega^{2}+\gamma^{2})}$$

is the imaginary part quantifying the relative phase shift of the induced polarization with respect to the external field, i.e., it determines the bandwidth and includes losses such as ohmic heat loss. Under the resonance condition, the extinction coefficient is maximum when $\varepsilon_{m}(\omega )=-2\varepsilon$ the NP radiates light with the characteristic of the dipolar plasmon. The Drude model suggests that the LSPR absorption is determined by both the dielectric function of the plasmonic materials $\left(\varepsilon_{m}\right)$ and the surrounding medium $\left(\varepsilon_{s}\right)$

(5) $$\varepsilon_{i}=\frac{\omega_{p}^{2}}{\omega \left(\omega +i\gamma \right)}$$

Two main mechanisms for SERS are (a) electromagnetic (EM) field where a Raman scattering molecule in the vicinity of the PNP is subject to the intense external field, which causes plasmon oscillation and, consequently, charge polarization. The plasmon field couples and amplifies the local electric field at the frequency ω on the surface of the PNP via LSPR. The higher electric field intensity results in strong polarization of the molecule, and thus, the higher induced dipole moment and intensified light absorption and scattering at the frequency of the SPR is obtained [24]. The total detected $I_{SERS}$ is

(6) $${\bar{E}}_{T}(r,t)={\bar{E}}_{1}(r,t)+{\bar{E}}_{2}(r,t)={\left|{\bar{E}}_{T}(r,t)\right|}^{4}$$

where ${\bar{E}}_{1}(r,t)=({\bar{E}}_{i}+{\bar{E}}_{s\;})$, ${\bar{E}}_{i}$ and ${\bar{E}}_{s\;}$ are the incident and elastic scattered light from the NP, respectively, and ${\bar{E}}_{2}(r,t)=({\bar{E}}_{\mu_{d}}+{{\bar{E}}^{\prime }}_{s})$ where ${\bar{E}}_{\mu_{d}}$ and ${{\bar{E}}^{\prime }}_{s}$ represent the induced dipole electric field and plasmonic scattered light because of LSPR [25]. Consequently, the energy couples to the plasma, which creates a frequency shift because of the energy difference between the frequency of the incident light and the molecular vibration. As a result, when the NP-molecule complex is excited, Raman scattering occurs, defined by $E_{R}=\beta_{R}E_{i}{\bar{E}}_{p}$ where $\beta_{R}$ is the fraction of photons undergoing inelastic scattering, $E_{i}$ is the incident field, and ${\bar{E}}_{p}$ is the average field enhancement over the NP surface (i.e., the ratio of the enhanced field to the incident field), (b) the second mechanism is a chemical enhancement due to charge transfer between molecule and NP where the molecules of the target analyte are chemically bound to surface of NP.

Among a variety of nanostructures, GNU is considered an anisotropic 3-D nanocrystal with different optical properties in comparison to spherical gold NPs of the same core size. This is because of the multi-branched shape and irregular surface morphology with various lengths, which results in redshifts within the SPR peak. A single GNU has several plasmon resonances (PRs) associated with individual tips, which contributes to a significant amplification of the EM field at the branch tips inside smaller and highly localized volumes [26]. Thus, the spectral and spatial localization of the PRs facilitates the selective excitation of various individual plasmonic hotspots in a nanoscale volume. As a result of the enhanced localized field, strong fields correspond to a large local density of states that leads to an equally strong enhancement of light-matter interaction at a spatial scale comparable to the size of the hotspots [27,28]. Therefore, the spiky core-tip structures allow more efficient biosensing or therapeutic applications due to a plethora of hotspots and a greater degree of functionalization with targeting species [29,30]. The development of an efficient label-free SERS probe for selective, rapid, and ultrasensitive detection of low abundance biomarkers in cancer diagnostics can be obtained by surface functionalization and bioconjugation of the NPs using a range of appropriate targeting agents, including mAb, aptamers, peptides, and cell surface ligands [31,32,33,34,35,36]. However, to ensure maximum efficiency, long-term stability of mAb or other ligands, and scalability, immunosensors are typically fabricated on solid substrates.

Following our recent works on the directional orientation of mAb on thin gold film [37] and colloidal GNU [38] for the detection of biomarkers, we propose to extend our research to fabricate plasmonic-active nanostructured thin film (PANTF) based on PCB. The advantages of PANTF are a higher sensitivity because of the coupling of the SPs of thin film and that of NPs and can be used as a transducer for different types of plasmonic-based sensing techniques: propagating SP, localized PR, and SERS-based sensing method [39]. Our system is comprised of 50 nm thick gold film deposited on the PCB and then functionalized hexane for conjugation of GNU, where the gap between the GNU and the film can also lead to reproducible hot spots [40]. It is then further functionalized by ADH for the directional orientation of mAb on the GNUs for more efficient binding of the target molecules compared with randomly oriented antibodies [36,38]. This, in turn, will increase the sensor efficiency for detection of the analyte molecules. Therefore, effectively, the PANTF SERS biosensor offers overall better performance than thin film and NP-immobilized substrate alone because of several field enhancements from both the GNU-layer and interparticle plasmon coupling (hot gaps) and, second, the high localized field at the tips [40]. Therefore, the goals of this research are: (a) Fabricate PCB-based TGF, (b) Covalent immobilization of GNU on PCB-based TGF, (c) Directional conjugation of mAb on GNU via ADH, (d) FT-NIR and SERS study of 2+, 3+, and Pos BCS biomarkers, and (e) Principal Component Analysis (PCA) of experimental data.

## 2. Materials and Methods

Deionized water (DI), reagent alcohol (277649), toluene, 2-propanol, 1,6-Hexanedithiol (HS(CH_2_)_6_SH, HDT, H12005, and phosphate-buffered saline (PBS, pH 7.4, Gibco, Thermo Fisher, Waltham, MA, USA), sodium chloride (NaCl, MW = 58.44 g/mol, sodium phosphate (NaH_2_PO_4_), hydrochloric acid (HCl), sodium periodate (NaIO_4_, MW = 213.89 g/mol), and ethanolamine (NH_2_CH_2_CH_2_OH, MW = 61.09 g/mol) were purchased from Sigma-Aldrich, Oakville, ON, Canada. Ethanol (70%) and ultrapure water were purchased from Thermo Fisher MA, USA. Citric acid stabilized 20 mL GNU with 90 nm diameter, 5.37 × 10^9^ NP/mL, weight concentration of 3.97 × 10^−2^ mg/mL, and molar concentration of 8.92 × 10^−12^ dispersed in 0.1 mM phosphate buffered saline was purchased from Cytodiagnostics, Burlington, ON, Canada. Bovine serum albumin (BSA, A2153-10G, Sigma-Aldrich, Oakville, ON, Canada) with an isoelectric point between 4.5 and 4.9 were used as the matrix solvent and the solution protein, respectively. The PEG linker chosen for functionalization was 5 kDa SH-PEG-COOH (HE003019-5K, Biochempeg, Watertown, MA, USA). Conjugation was performed using HER-II mAbs, as shown in Table 1. Low retention 1.5 mL microcentrifuge tubes (3451PK, Biolynx, Brockville, ON, Canada) were used as reaction tubes. Equipment used in the reaction process was a microcentrifuge (OF-17710-11), a centrifugal filter (UFC503096, Millipore Sigma, Burlington, ON, Canada), an orbital shaker (RK-51700-13), and a vortex shaker (RK-04729-07) purchased from Cole-Palmer, Quebec, QC, Canada, a combo pH meter BLU2300E), an analytical balance (Sartorius, Oakville, ON, Canada ), as well as micropipettes and micropipette tips (Eppendorf, 2231302001). 2-(N-Morpholino) ethane sulfonic acid hydrate (MES hydrate, M8250-25G), sodium hydroxide (NaOH, 72068-100ML), and N-(3-Dimethylaminopropyl)-N′-ethyl carbodiimide hydrochloride (EDC, E7750-10G), and adipic acid di-hydrazide (ADH) were purchased from Sigma-Aldrich. Hydroxysulfosuccinimide (sulfo-NHS, 24510) and ultrapure water (10977023) were purchased from Thermo Fisher. Three types of HER-II breast cancer serums (BCS) were provided for the interaction study, see Table 1. Mixing some toxic solution was performed inside a fume hood (ISOLA, Mystaire, Creedmoor, NC, USA).

### 2.1. Preparation of PCB Substrate

**Figure 1:**
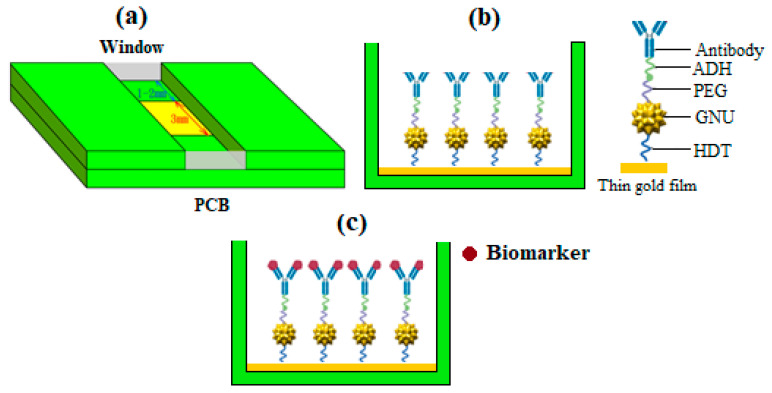
Schematic representation of (**a**) thin gold film-coated PCB channel enclosed by windows, (**b**) functionalized and directional mAb-conjugated GNU plasmonic sensor, and (**c**) plasmonic PCB-nanostructured sensor capturing biomarkers during the interaction process.

Three PCB substrates were prepared (i.e., one for each BCS). Figure 1a indicates an example of a PCB designed and fabricated for the experiment. The nickel component forms a barrier against copper diffusion and protects against contamination. The final product is highly resistant to corrosion. A thin gold layer with a thickness of 50 nm was coated on a PCB base using an electroless deposition in a single-step bath immersion technique. Electroless deposition or plating of metals is a uniform coating of a metallic layer, such as gold, on the surface through the chemical reduction of metal ions in an aqueous solution and the subsequent deposition of metal without the use of electrical energy. The bath produces a much tighter, lower porosity deposit.

#### 2.1.1. Initial Rinsing

The substrates were initially cleaned by rinsing with 70% ethanol (in the squeeze bottle, (BP82031GAL, Fisher Scientific, Ottawa, ON, Canada) to remove any Kimwipe/substrate particulates. It was placed in a small circular container where 70% ethanol was squeezed into the channel, removed by Kimwipe after 30 s, and left to air dry.

#### 2.1.2. Channel Enclosure

Prior to the GNU immersion immobilization, functionalization, and conjugation, both ends of the channel of each PCB had to be closed. Two microscope glass slides were cut with dimensions of approximately 5 × 15 mm, filed, and cleaned using ethanol-soaked Kimwipe before adhering them to the sides of the channel. The channel was tested for leakage using DI water.

#### 2.1.3. Final Sonication

The substrates were placed inside a plastic container, and 500 μL of 70% ethanol was added to the channel for the purpose of cleaning. The container was sealed and sonicated (Elma, Singen, Germany) at 60 kHz and 60% power for 30 min to clean the gold surface thoroughly. An ethanol rinse was repeated twice to ensure the surfaces were clean. The substrates were air-dried and stored at room temperature until further use.

## 3. Fabrication of PCB Sensor

### 3.1. Functionalization of PCB

#### 3.1.1. Preparation of 15 pM GNU

Inside the biosafety cabinet, the volumes of 8.92 pM supplied 90 nm GNU were added to three separate microcentrifuge tubes: 1.12 mL (tube I), 1.12 mL (tube II), and 1.13 mL (tube III). The tubes were microcentrifuged at 3200 rpm for 30 min. Once centrifugation was complete, the supernatant of each tube was removed, leaving behind 25 μL of pellet from each tube. Ultrapure water was added to the three GNU-containing tubes: 0.56 mL to tubes I and II and 0.565 mL to tube III, and the pellets were resuspended with a micropipette. The entire volumes of GNU in tubes I, II, and III were transferred to a clean 20 mL glass scintillation vial for a total of 1.685 mL of resuspended GNU. To this vial, 0.35 mL of ultrapure water was added for a final volume of 2.035 mL of 15 pM GNU to use for coating the PCB substrate.

#### 3.1.2. Preparation of HDT

In the biosafety cabinet (Nuair Labgaurd ES Class II), 1999 μL of pure ethanol reagent alcohol and 1 μL of HDT were mixed in a 20 mL glass scintillation vial and capped immediately. The vial was vortexed at 1800 rpm, creating a 2 mL solution of 3 mM ethanolic HDT.

#### 3.1.3. Preparation of 5 mM PEG Linker Solution

Inside the biosafety cabinet, 50 mg of 5k HS-PEG-COOH was weighed on the analytical balance and added to a clean 20 mL glass scintillation vial containing 2 mL of ultrapure water. The vial was mixed by vertexing at 1800 rpm for 30 s to ensure the PEG had dissolved.

#### 3.1.4. Functionalization of PCB with HDT and GNU

Clean PCB-coated gold substrates were obtained and placed in a clean container. 200 μL of 3 mM ethanolic HDT was pipetted on the gold surface, and the container was capped and placed on the orbital shaker for 2 min at 150 rpm. The substrates were immersed in the 2 mL of 3 mM HDT vial and then sonicated at 60 kHz and 60% power for 2 min to functionalize the gold surface with HDT molecules. After 2 min, the excess HDT was rinsed with 70% ethanol and ultrapure water inside the biosafety cabinet. Immediately after the rinse, 200 μL of prepared 15 pM concentrated GNU was pipetted onto the gold surface. The container was then sealed and placed on the orbital shaker for 10 min at 150 rpm. The substrates were then rinsed with ultrapure water and air-dried in the biosafety cabinet.

#### 3.1.5. Functionalization with PEG

The GNU-functionalized PCB substrates were further functionalized with the prepared 5 mM-COOH terminated PEG linker. This was achieved by adding 200 μL of PEG linker to each of the four channels. The chambers were then capped and left to mix overnight on the orbital shaker at 150 rpm at room temperature. The next morning, the substrates were rinsed with ultrapure water to remove any free PEG.

#### 3.1.6. Functionalization with ADH

Before measuring any chemicals, the containers were brought to room temperature. On the analytical balance, 58.2 mg of ADH and 29.1 mg of (EDC) were measured. Both powders were added to a clean 20 mL glass scintillation vial containing 2.0 mL of ultrapure water and were dissolved by vertexing at 1800 rpm for final concentrations of 167 mM ADH and 76 mM EDC. To each of the substrates, 200 μL of the prepared ADH/EDC solution was added to fill the channel depth, and they were left to mix on the orbital shaker at 150 rpm for 3 h. Once the time was complete, the substrates were rinsed with ultrapure water to remove excess reagents. PEG is a water-soluble biocompatible polymer which binds to water via hydrogen bonding. It has thiol (-SH) and carboxyl (-COOH) functional groups in its terminal ends. Sulfur attaches to GNU via gold-thiol bonds, while the carboxyl group interacts with the hydrazide functional group on ADH (-CONH-NH_2_) to form a stable hydrazide bond (-CONHNH-).

### 3.2. Conjugation of mAb

The mAb was thawed overnight in the fridge, supplied as 200 μL at a 1 mg/mL concentration. Each substrate requires 25 μg of mAb, so a total of 50 μL of mAb was buffer exchanged. To complete the buffer exchange, the supplier buffer of the mAb had to be exchanged with the newly prepared 100 mM sodium phosphate buffer. To accomplish this, two 30 kDa centrifugal filters were used.

#### 3.2.1. Preparation of Coupling Buffer

A fresh “coupling” buffer had to be prepared for the activation and purification of mAb. The coupling buffer consists of 10 mM sodium phosphate, 0.15 M sodium chloride, pH 7.5. To prepare 10 mL of the buffer, 6 mL of distilled water was added to a clean 20 mL glass scintillation vial. Then, 14.02 mg of NaH_2_PO_4_ and 87.68 mg of NaCl were measured and added to 8 mL of water. The initial pH was measured as 8.6. To lower the pH to 7.5, first, a diluted HCl was prepared by adding 5 µL of 37% HCl to 4 mL of distilled water, then adjusted by adding 1.6 mL of dilute HCl to the 8 mL of buffer in the glass beaker. After the pH reached 7.5, the buffer was transferred to a graduated cylinder, and distilled water was added to the cylinder until the final volume reached the 10 mL mark. The buffer was then stored in the fridge for further use.

#### 3.2.2. mAb Activation and Purification

The mAb was activated by preparing 0.1 M NaIO_4_ solution, where 2.14 mg of NaIO_4_ was added to 100 µL ultrapure water in a clean 1.5 mL microcentrifuge tube. The NaIO_4_ tube was mixed and wrapped in aluminum foil to protect it from light. Then, 20 µL of the prepared 0.1 M NaIO_4_ solution was added to the tube containing 100 µL of mAb. The tube was vortexed at 2200 rpm for 30 s and then incubated at room temperature for 45 min at 275 rpm on the orbital shaker. After incubation, the reaction was quenched by adding 300 µL of PBS to the tube of mAb. At this stage, the existing mAb buffer was exchanged for the new coupling buffer prepared in the previous step using the spin columns. The similar steps as in the previous buffer exchange were repeated using the entire volume of mAb (~420 µL). The final recovery buffer used for the reverse spin was 100 µL of coupling buffer. The final mAb volume was 100 µL at 1 mg/mL.

#### 3.2.3. Reaction Incubation

A volume of 25 µL of 1 mg/mL mAb was added from the 100 µL of previously activated mAb. In addition, 100 µL of coupling buffer was added to each substrate for a total reaction volume of 150 µL on the active area. The chambers were separately sealed and left on the orbital shaker overnight at room temperature at 150 rpm.

#### 3.2.4. Hydrazone Bond Stabilization

To stop the hydrazone bond (i.e., the bond between ADH-mAb) from reversing, the reactions were stabilized by the addition of 2 µL of 1M ethanolamine inside the fume hood using acid gloves and proper PPE. A volume of 2 µL of ethanolamine was added to each reaction and incubated overnight. The reactions were again incubated at room temperature at 150 rpm on the orbital shaker for 1 h.

#### 3.2.5. Blocking

A 10% *w/v* BSA solution was prepared by adding 6.0 mg of BSA to 60 µL of coupling buffer in a clean microcentrifuge tube to block the surface of the nanocomplex. Then, 15 µL of the BSA was added to each of the reactions. The tubes were then incubated at room temperature for 10 min at 150 rpm on the orbital shaker. The excess BSA was washed off with PBS, and the conjugation of mAb in the substrates was now completed. The pH environment was kept at an optimal value at pH 6 throughout the experiment, reducing the likelihood of interference. Figure 1b illustrates the completed mAb-conjugated PCB-based nanoplatform for sensing the biomarker.

#### 3.2.6. Healthy Blood Serum (HBS)

The mAb used was 25 µg of anti-HSA (MAB1455, R&D Systems, Canada), which was thawed overnight prior to use. While the mAb reaction tube was incubating, the BSA-blocking solution was prepared according to the procedure outlined above. In the biosafety cabinet, the pooled type-AB Healthy Blood Serum (HBS, ISERABHI100ML, Innovative Research, USA) was diluted in the prepared 0.01 M PBS buffer (pH 6.0) in a clean 1.5 mL microcentrifuge tube (8 µL HBS: 112 µL PBS buffer). To this dilution, 280 µL of MES (pH 6.0) was added to make the total volume of serum dilution 400 µL. From the prepared serum dilution, 200 µL was added to the surface of each of the functionalized PCB substrates. The chambers were sealed and incubated at room temperature in the dark at 150 rpm on the orbital shaker for 1 h. After incubation, the excess reagents and unbound serum proteins were rinsed off with the same 0.1 M MES buffer. The PCBs were air-dried before characterization.

## 4. Interaction of mAb-BCS

### 4.1. Serum Dilution

The BCS was diluted in the biosafety cabinet using 0.01 M PBS buffer (pH 6.0) and a clean 1.5 mL microcentrifuge tube (16 µL BCS: 224 µL PBS buffer). To this dilution, 400 µL of MES (pH 6.0) was added to make the total volume of serum dilution 640 µL. The BCS dilution was vortexed at 2200 rpm for 30 s to mix and then divided into three PCB substrates.

### 4.2. Incubation

For the incubation, 160 µL of prepared serum dilution was introduced to the surface of the three functionalized PCB substrates. The chambers were then sealed, covered, and incubated at room temperature at 150 rpm on the orbital shaker for 1 h. After incubation, the excess reagents and unbound serum proteins were washed with the same 0.1 M MES buffer. Figure 1c shows the sensor after capturing the BCS (HER-II) biomarker.

A common method of bioconjugation is to activate carboxylic acids using EDC and N-Hydroxysulfosuccinimide (sulfo-NHS) where EDC couples NHS to carboxyl’s, forming an NHS ester that is considerably more stable than the O-acylisourea intermediate while allowing for efficient conjugation to primary amines (i.e., mAb) to form an amide at physiologic pH. The conventional EDC/sulfo-NHS activation technique enables the conjugation of the mAb to carboxylated linker molecules such as PEG. Therefore, PEG-functionalized GNU can be linked with mAb targeting epitopes within the intracellular domain of HER-II receptor proteins, serving as biomarkers for BC. The targeted orientation of the mAb on the PEGylated surface of GNU using ADH enhances selectivity significantly and improves accessibility to the antigen-binding region, in contrast to conventional EDC/sulfo-NHS conjugation techniques. The specific alignment of the mAb on the gold surface involves activating the glycosylated oligosaccharide moieties within the constant (Fc) region of the mAb using sodium periodate. The activation of the glycosylated oligosaccharide moieties in the constant (Fc) region of the mAb with sodium periodate achieves the selective orientation of the mAb on the gold surface.

The maximum orientation is obtained via the lower part of the Fc, which is ideal for immobilization. Following activation, the Fc regions can establish exceptionally stable hydrazone bonds with ADH molecules, facilitating precise orientation of the mAbs while keeping the antigen-binding sites available for binding. The primary amine (-NH_2_) groups crucial in EDC/sulfo-NHS chemistry methods are distributed across all regions of the mAb, enabling its binding to a carboxyl-functionalized (-COOH) surface in multiple orientations. The antigen-binding regions, specifically the variable Fab region, feature primary amine groups that are susceptible to amide bond formation. Consequently, these regions become unavailable for antigen binding [41,42]. Because of the strong affinity between -SH groups and gold, high-density single-chain variable fragments can be adsorbed in a site-specific manner on the gold-coated substrate. This facilitates the more efficient detection of the biomarker using the EDC/ADH hydrazone bond chemistry method. This occurs because of the selective bond formation at the Fc region, enhancing the accessibility of the Fab region that mediates antigen binding, specifically in the e‘nd-on’ position. Furthermore, the enhancement in light scattering properties is attributable to the plasmon component perpendicular to the surface, which correlates with the molecular polarization oriented vertically relative to the surface [42]. Therefore, at low concentrations, proteins on the gold surface typically align their aromatic amino acid rings parallel to the surface plane. However, as the surface approaches monolayer coverage, these rings tend to stack with their planes oriented perpendicular to the surface.

## 5. Characterization

**Figure 2:**
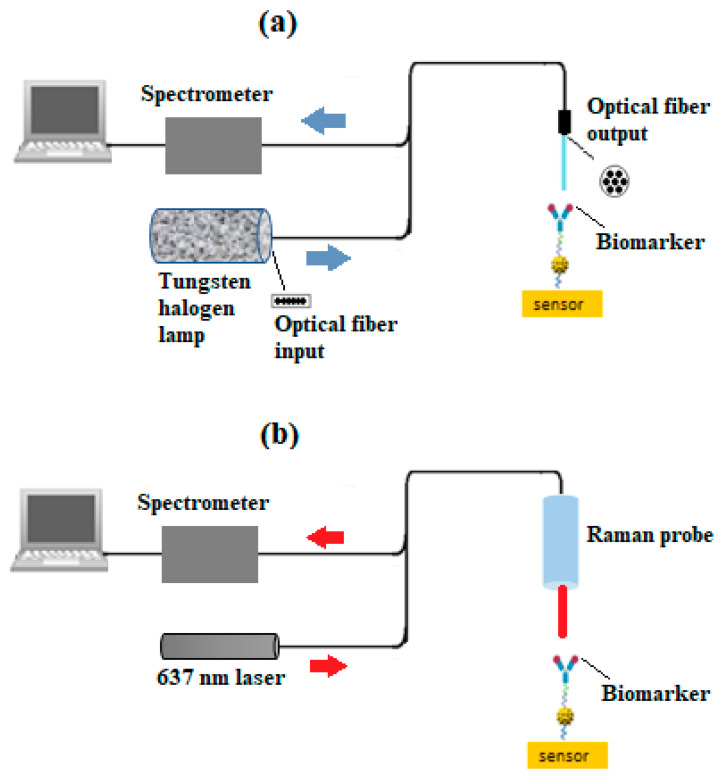
Experimental setup for (**a**) FT-NIR and (**b**) SERS.

The surface morphology of the PCB before and after functionalization is studied by SEM and AFM, and it can be used in different operating modes so that one can study the roughness or morphology of the surface on different scales. In this approach, the displacements of the surface points from the reference plane are modeled as a random surface and characterized by appropriate distribution of the height function (roughness) values *z* [43]. The absorbance was measured using a UV-vis spectrometer (Jenway 7205-Cole-Parmer, Quebec, QC, Canada) covering a spectral range from 198 to 800 nm. Figure 2a illustrates the FT-NIR setup in reflectance mode, where the PCB was analyzed using an FT-NIR Nano Quest spectrometer (NanoQuest 2.5, Ocean Insight (Gamble Technology), Mississauga, ON, Canada). The spectrometer was configured with a 5-s integration time and 8 nm resolution. A 200 µm bifurcated reflectance probe (RP28, Thorlabs, Saint-Laurent, QC, Canada) was employed, with one leg connected to a tungsten halogen source (HL-2000-HP, Ocean Insight, (Gamble Technology), Mississauga, ON, Canada), delivering light to the sample at a perpendicular distance of 10 mm. The other leg delivered the reflected light to the spectrometer for analysis. The background reading was a solid white foam surface. Figure 2b illustrates the SERS setup, where a 638 nm Raman probe (RIP-RPB-638-FC-APC-SMA, Ocean Insight, (GambleTechnology), Mississauga, ON, Canada) was positioned perpendicularly to the PCB surface at a distance of 7.5 mm (the focal distance of the probe). The probe was connected to a 637 nm laser operating at 5 mW (S1FC637, Thorlabs, Saint-Laurent, QC, Canada) with a spot size of ≈1 mm, and an Ocean HDX spectrometer (OCEAN-HDX-VIS-NIR, Ocean Insight, Orlando, FL, USA) was utilized. Parameters in Oceanview software were configured as follows: 5 s integration time, averaging of three scans, and a boxcar width of 5. Nonlinearity correction was applied, and the clean peaks option was selected. A background measurement was taken with the 637 nm laser turned off.

## 6. Results and Discussion

### 6.1. SEM

**Figure 3:**
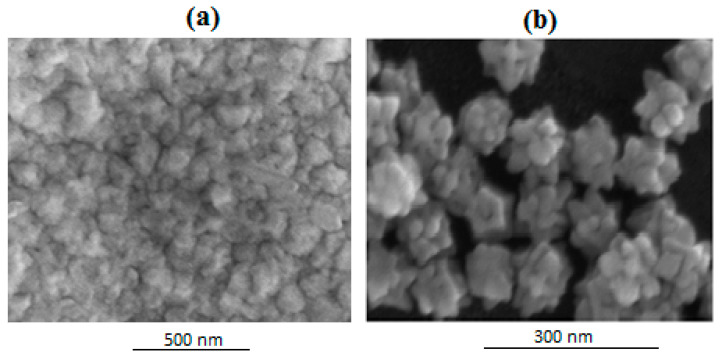
SEM images of GNU-immobilized substrate at (**a**) lower and (**b**) higher magnifications.

Figure 3a indicates the SEM image of GNU-immobilized substrate by immersion technique where the surface is covered by a high-density number with some degree of agglomeration or clustering. It is seen more clearly in Figure 3b at a higher magnification that some clustering occurs while other GNU forms a monolayer. It is noteworthy that some regions are void of GNU, which can affect the efficiency of the results.

### 6.2. AFM

**Figure 4:**
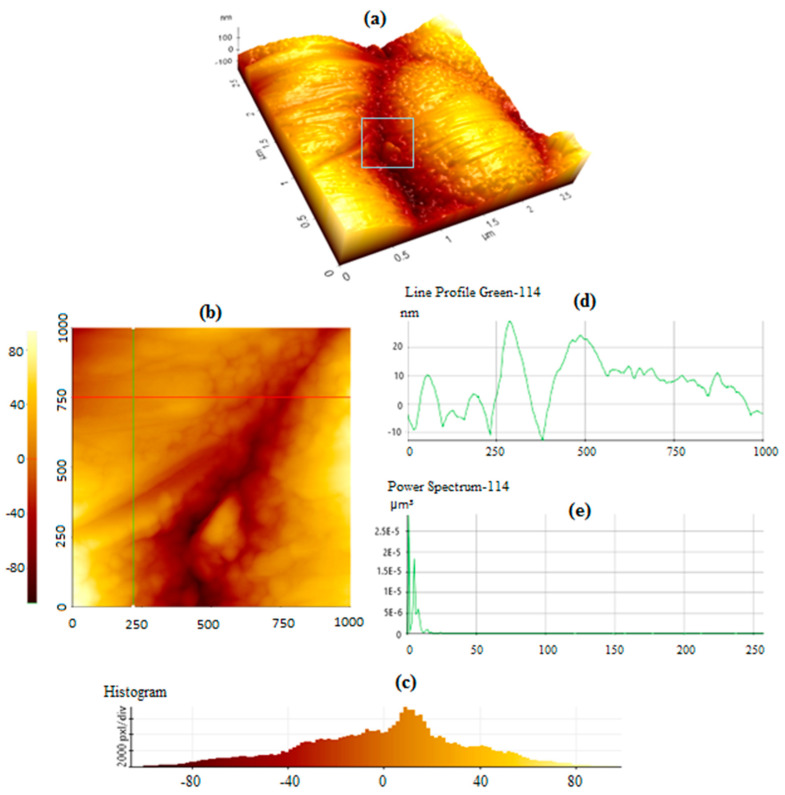
AFM image of (**a**) the PCB before functionalization, (**b**) closeup of the surface, (**c**) histogram, (**d**) line profile, and (**e**) power spectrum of the fabricated sample.

Figure 4a shows an AFM image of the fabricated TGF-coated PCB bumpy surface prior to GNU immobilization. Figure 4b is a magnification of the inset in Figure 4a with the corresponding histogram of the surface topology given in Figure 4c. Figure 4d shows the line profile, which describes a 2-dimensional tolerance zone around any line in any feature, usually of a curved shape, and allows measurement of the depth and the width of the mark. In this case, a valley with a depth of −10 nm and a height of ≈30 nm is observed. It is noteworthy that dissimilar to the line profile, which describes a specific cross-section on the part, the profile of a surface controls every cross-section across the entire length of the surface. The power spectrum shown in Figure 4e provides both lateral and vertical signals captured from AFM images.

Asymmetry of the roughness distribution can be tested by using the sample coefficient of $R_{sk}$ [44]:

(7) $$R_{sk}=\frac{\sum_{i=1}^{n}{\left(z_{i}-\overset{-}{z}\right)}^{3}}{ns^{3}}$$

where $R_{sk}$ is the skewness, or measure of symmetry over the surface profile, i.e., the lack of symmetry around the data point distribution curve, n is the number of height function values, z is the sample mean, and s is the sample standard deviation and is defined as follows:

(8) $$s=\sqrt{\iint {\left(f\left(x,y\right)\right)}^{2}dxdy}$$

**Figure 5:**
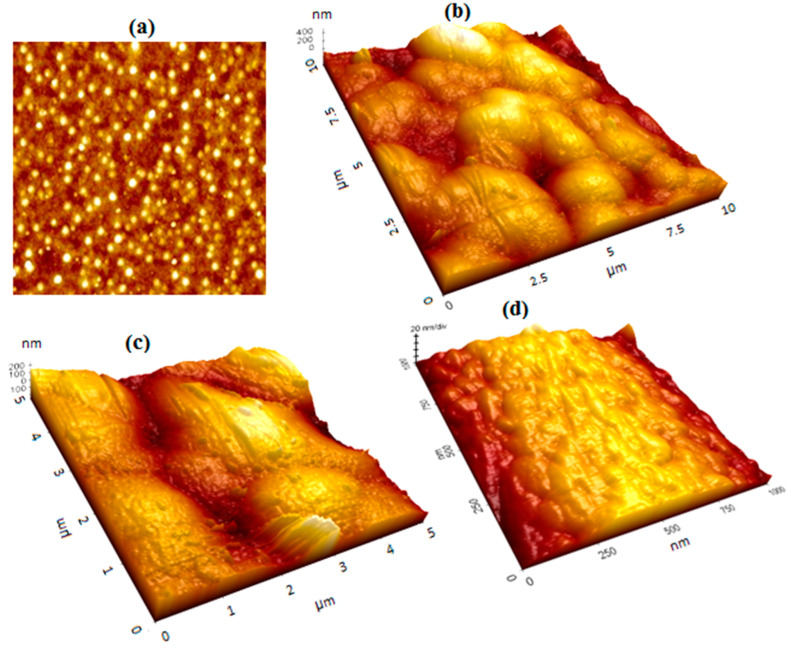
(**a**) Top and (**b**–**d**) side views of the GNU-immobilized substrate at increasing magnification.

Other parameters are the root mean square average of height deviation ($R_{q}$) taken from the mean image data plane and the arithmetic average of the absolute values of the surface height deviations ($R_{a}$) measured from the mean plane. Based on experimental data and the software, values of 9.011 nm, 7.147 nm, and −0.173 are obtained for $R_{q}$, $R_{a}$, and $R_{sk}$, respectively. Large values of *|R_sk_|* implicate deformation of the roughness distribution shape. Negative skewness indicates the predominance of deep, narrow valleys, and positive skewness denotes high, narrow peaks, i.e., a ripple-type surface, as illustrated in Figure 5. Figure 5a is a top view of the sample where the GNUs are distributed at the surface, some dispersedly and some closely agglomerated. Figure 5b–d indicates the surface topology at increasing magnifications.

**Figure 6:**
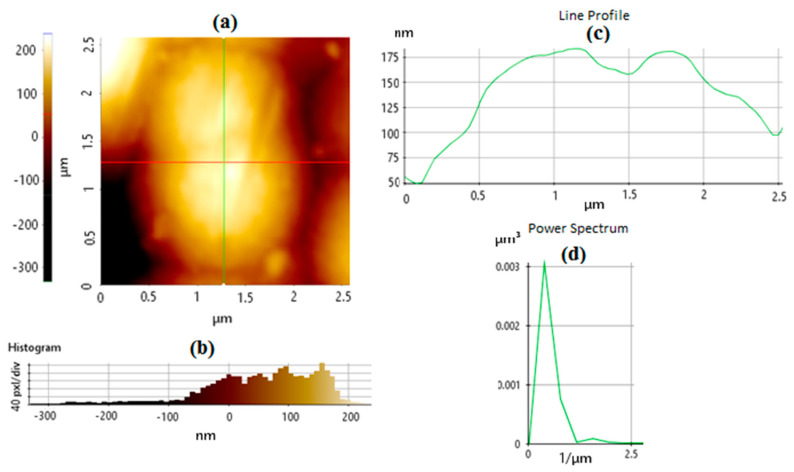
(**a**) A closeup AFM image of the PCB after functionalization, with the corresponding (**b**) histogram, (**c**) line profile, and (**d**) power spectrum.

There is a distinct difference in the texture of GNU-immobilized PCB compared with untreated PCB In Figure 4. However, there is a relatively weak contrast between these two because of the background optical and material similarities, which might be improved if AFM is used in contact mode at the expense of minimally scratching the surface. Figure 6a represents the surface of PCB after GNU immobilization with the corresponding histogram in Figure 6b, line profile in Figure 6c, and the power spectrum in Figure 6d. The aforementioned discussion also applies in this case, except that the line profile indicates a positive value of height because of the presence of a 90 nm single and possibly agglomerated GNUs at the surface. Using the AFM software, the values of $R_{q}$ = 39.405, $R_{a}$ = 33.045, and $R_{sk}$ = 0.914 are achieved. Note that the power spectrum in this case shows a higher value than without GNU, i.e., higher lateral and vertical signals captured from the AFM images.

### 6.3. Enhancement Factor

**Figure 7:**
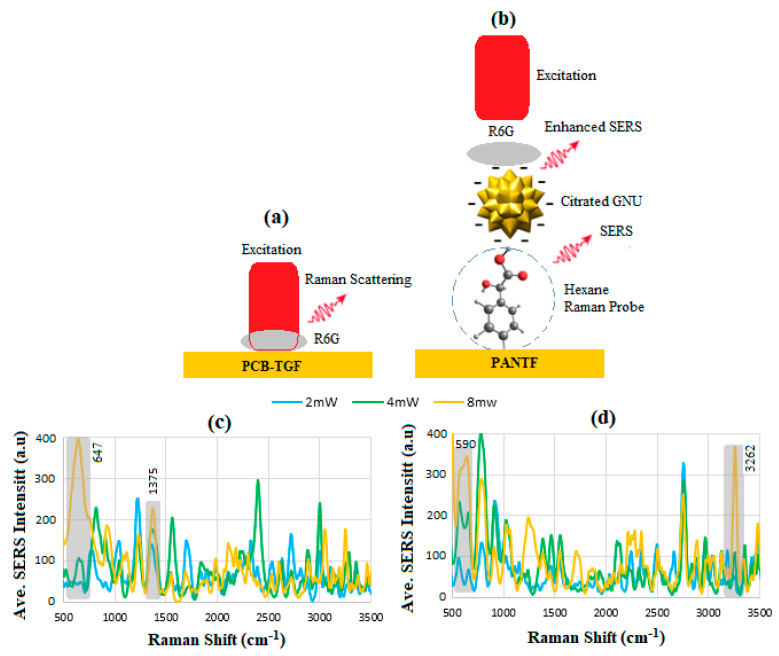
Schematic representation of R6G dye SERS for (**a**) PCB-TGF, (**b**) PANTF, and averaged SERS intensity of R6G for (**c**) PCB-TGF at 10 mM, and (**d**) PANTF at 1μM after 10 min GNU incubation via thiol covalent bond, respectively.

Figure 7 illustrates the experimental setup for the SERS study of PCB-TGF and PANTF using R6G dye, shown in Figure 7a and Figure 7b, respectively. To determine the EF of the sensor, 10 μL of 10 mM and 1 μM of R6G as the Raman active probe molecule were added to the PCB-TFG and PANTF. In the case of PANTF, 10 μL of R6G accounted for the greater surface area of the GNU during the SERS reading. The corresponding SERS for PCB-TGF at various laser powers is shown in Figure 7c, where the overlapped peaks, such as 647 cm^−1^ and 1375 cm^−1^, correspond to the C-C-C ring in-plane vibration mode and C-C stretching of the aromatic ring, respectively. Similarly, Figure 7d indicates the SERS for PANTF, where the peaks between 590 and 600 cm^−1^ and >2800 correspond to in-plane bending vibration and NH stretching modes, respectively. It was noticed that the NH_3_^+^ I amine peak exhibited an enhancement in the presence of GNU versus without. However, the other R6G peaks corresponding to benzoic ester and secondary amine functional groups did not experience the same degree of enhancement. It is hypothesized that the NH_3_^+^ functional group’s high enhancement is because of the electrostatic interaction between its positive charge and the inherent negative charge on GNU. At the overlapped positions, the peak intensity increases systematically by increasing the laser power. In addition, the fluctuation of the intensities is thought to be due to a variety of charge transfer contributions likely induced by dynamical changes in the molecule environment connected to a random walk of the molecule onto the metallic surface during the adsorption process [45,46].

It can be discussed that the difference between the spectra in Figure 7a can be due to the variation in scattered light at various power levels, hence the intensity $I={\left|\bar{E}\right|}^{2}$ where $\bar{E}$ is the electric field and/or the heating effect on the molecules at the microscale such as thermally-induced fluctuation or diffusion of or dynamical changes in microenvironment, which according to quasi-classical thermodynamic can be stochastic in nature. However, in the case of Figure 7b, the orientation of the analyte molecules on the surface of the GNU is also crucial as the scattering originates from the component of the LSPR perpendicular to the surface, which is related to the molecular polarization vertical to the surface. Therefore, the coupling between electronic states and vibrational modes of the R6G molecules and small changes in the position of adsorbed molecules on the TGF or GNUs can create a temporal modulation of these states responsible for the enhancement of molecules. The intensity of lines enhanced due to LSPR is used to determine the EF at a given power using Equation (9) [47]

(9) $$EF=\frac{I_{SERS}}{I_{Raman}}\times \frac{C_{Raman}}{C_{SERS}}$$

where $I_{Raman}$ is the intensity of bare substrate with R6G at concentration $C_{Raman}$ = 10 mM, $I_{SERS}$ is the intensity of GNU-immobilized substrate with R6G at concentration $C_{SERS}$ = 1 μM, and $\frac{I_{SERS}}{I_{Raman}}=\frac{\langle \approx 300\rangle }{\langle ≃115\rangle }$, so $\frac{C_{Raman}}{C_{SERS}}$ = 10^4^. Therefore, by using the above values, $EF=2.6\times 10^{4}\approx 0.3\times 10^{5}$ was obtained.

### 6.4. UV-Vis Spectroscopy

**Figure 8:**
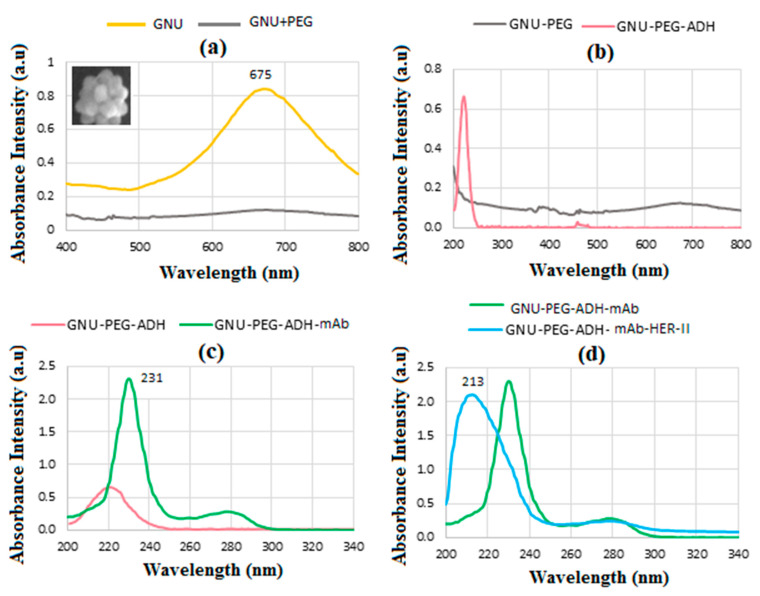
UV-vis absorbance of the bioconjugates at various stages of fabrication. (**a**) GNU LSPR at 675 nm and after functionalization by PEG. The inset represents an example of isolated GNU, (**b**) functionalized by ADH showing a peak at 222 nm, (**c**) after functionalization of ADH with mAb where the peaks at 231 and 280 nm corresponding to Tyr and possibly Trp are observed, and (**d**) shows the HER-II interaction with mAb with a peak at 213 nm.

Figure 8 displays the UV-vis spectra depicting different stages of functionalization of the conjugate, beginning with GNU and culminating in GNU-PEG-ADH-mAb, the final step before biomarker detection. The GNU exhibits a distinctive SPR absorbance peak at approximately 675 nm, illustrated in Figure 8a. After surface functionalization of GNU with EG, the intensity of SPR absorbance decreases by approximately 80%. The peak at 222 nm in Figure 8b is due to ADH-functionalised PEG before the mAb conjugation. Following the conjugation of mAb, Figure 8c shows the emergence of two distinct peaks at 231 and 280 nm, which correspond to the aromatic amino acid side chains of tyrosine (Tyr) and tryptophan (Trp) [48]. In Figure 8d, the interaction between mAb and the HER-II biomarker is depicted, revealing two distinct peaks at 213 and 280 nm, corresponding to the phenylalanine (Phe) and try amino acids present in the biomarker, respectively.

### 6.5. FT-NIR Spectroscopy

The NIR spectral region ranges from 800 to 2500 nm (12,500–4000 cm^−1^) with absorptions representing overtones and combinations mainly associated with -CH, -OH, -NH, and -SH functional groups and can provide unique information covering from life sciences to environmental issues [49]. The NIR spectrum is divided into three regions of short-wave spanning from 800 to 1200 nm (12,500–8333 cm^−1^), represents bands resulting from electronic transitions, overtones (i.e., the bands that are multiples of the fundamental absorption frequency mainly consists of carbon), and combinations modes. Region II ranges from 1200 to 1800 nm (8333–5500 cm^−1^), which covers the first overtones of (C, O, N), stretching vibrations, and various types of combination modes. This range is useful for quantitative and qualitative analysis as well as applications to structural studies such as those of hydrogen bonds. Finally, Region III (1800–2500 nm or 5500–4000 cm^−1^) is a combination mode region that deals mostly with the combination modes. NIR spectroscopy is concerned with both electronic and vibrational transitions [50]. Due to electronic transitions, bands are observed in the NIR region and, in general, are presented as weak bands.

**Figure 9:**
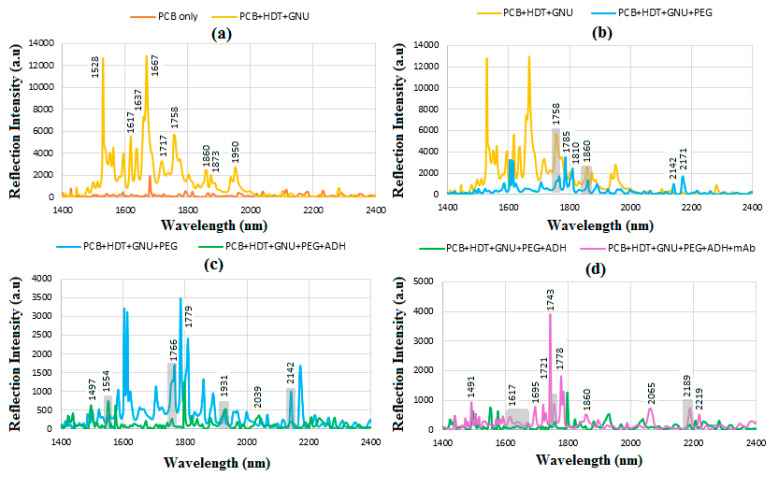
FT-NIR reflection signals for (**a**) PCB only and PCB-HDT-GNU, (**b**) PCB-HDT-GNU-PEG, (**c**) PCB-HDT-GNU-PEG-ADH, and (**d**) PCB-HDT-GNU-PEG-mAb.

Figure 9a illustrates the comparison of the reflective intensity for PCB, mainly between 1500 and 2000 cm^−1^ and PCB-immobilized GNU. The reflectivity significantly increases after immobilization with the dominant lines at 1528, 1667, 1758, and 1950 nm, respectively.

The intensity of reflection signals decreases significantly after PEG-ylation. No peaks are observed between 4000 and 4500 cm^−1^ (2.500–2.222 μm) related to C-H combination and CH_2_ bonds. The range between 4550 and 5000 cm^−1^ (2.200–2.000 μm), such as 4668 and 4719 cm^−1^ (2.189 and 2.119 μm), correspond to the O-H bonds followed by the O-H first stretch overtones between 5000 and 5500 cm^−1^. Most of the PEG lines are seen between 5500 and 6000 cm^−1^, i.e., 1.875–1.650 μm related to C-H first stretch overtone. The lines between 6000 and 7500 cm^−1^ (i.e., 1.666–1.333 μm) mainly correspond to the presence of O-H first stretch overtones, C-H combination overtone, and CH_2_ combination first overtone, respectively. In Figure 9b, the O-H first stretch overtones and C=O stretch 2nd overtone are seen between 5000 and 5500 cm^−1^, followed by the C-H first stretch overtone including the dominant reflections at 1.758, 1.785, and 1.810 μm corresponding to 5688, 5602, and 5525 cm^−1^, respectively. The lines between 6000 and 7500 correspond to the presence of O-H first stretch overtones, such as the lines at 1.6 and 1.860 μm, i.e., 6250 and 5376 cm^−1^, and CH_2_ combination first overtone, respectively. The peaks between 4000 and 4500 cm^−1^ are related to C-H combination and CH_2_ bonds, and the O-H bonds in the carboxyl group of the PEG linker are responsible for the observed signal between 4550 and 5000 cm^−1^ with strong lines such as 4606 cm^−1^ (2.171 μm).

Note that the reflections at 1.758 and 1.860 μm overlap with that of the PEG molecular bonds. Before conjugating mAb, the orientation of PEG on the GNU surface can be assessed by examining the detection level, distinguishing between top and bottom bonding. Top bonding refers to intermolecular bonds within the PEG chain extending towards the COOH end, while bottom bonding involves specific bonds such as C-S and S-Au near the GNU surface. Due to their simpler structure compared with mAb, PEG linkers provide practical insights into parallel and perpendicular bonding through in-plane and out-of-plane vibrations, facilitated in our case by ADH linkage. The tentative assignment of PEGylated GNU is detailed in Table 2 [50,51].

When PEG is functionalized with ADH in Figure 9c, the intensity considerably decreased as before, with the peaks at 1.554, 1.766, 1.921, and 2.142 μm corresponding to 6435, 5662, 5205, and 4668 cm^−1^ overlapped. Figure 9d displays the results following the conjugation of GNU-PEG with mAb, characterized predominantly by amide vibrations originating from the peptide bonds present in the protein structure. A notable example of a globular protein is γ-globulin and its subclasses, such as immunoglobulins (e.g., IgG), which consist primarily of 60–70% β-sheets with minimal α-helix structure [52,53]. In this context, the majority of peaks are observed between 1.49 and 2.2 μm (corresponding to 6711–4545 cm^−1^), indicative of α-helix, β-sheet, NH_2_ combination, amino acids, first overtone symmetric and antisymmetric OH stretching, C-H combination, and CH_2_ combination [54]. The provisional assignment of mAb-conjugated PEGylated GNU is outlined in Table 3 [51,55,56,57].

**Figure 10:**
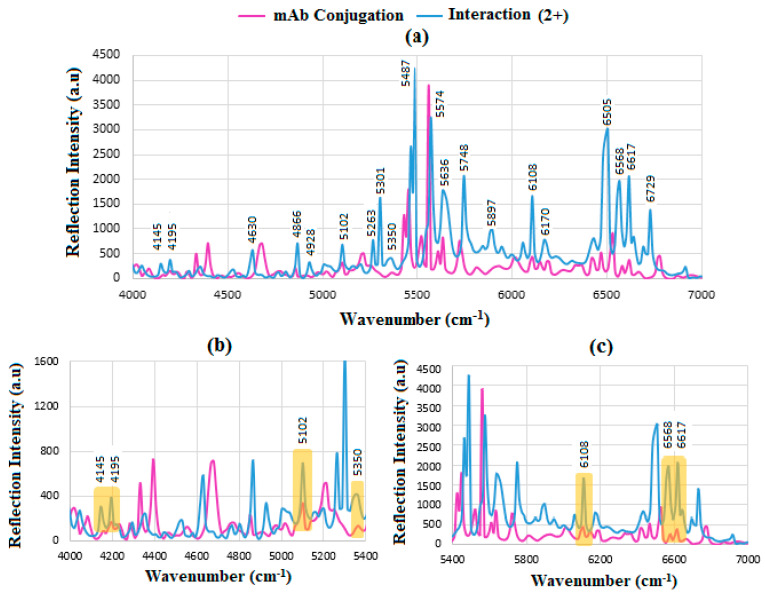
FT-NIR reflection signals for (**a**) before and after interaction of mAb with HER-II (2+), (**b**) the enlarged range between 4000 and 5400 cm^−1^, and (**c**) 5400–7000 cm^−1^ showing the areas of overlapped spectra corresponding to the presence of mAb and carbohydrate components.

Carbohydrate biomarkers, or sugar molecules, consist of carbon, hydrogen, and oxygen atoms (CHOs). These molecules serve as indicators of health or disease-related changes and are crucial in the diagnosis and treatment of conditions such as diabetes, cancer, and cardiovascular diseases. They are classified into monosaccharides (glucose and fructose), disaccharides (sucrose), polysaccharides (starch and fructus), oligosaccharides (raffinose), and sugar alcohols (inositol, sorbitol, and mannitol) [58,59]. Figure 10a demonstrates the mAb conjugation before and after interaction with BCS (HER-II-2+), where the intensity of reflection increases, indicating the binding between mAb and the biomarker. The spectrum is divided into parts to visualize the peaks more easily by magnifying the range. The strips in Figure 10b,c indicate the regions where the mAb and the biomarker overlap, which can be used as indicators of biochemical binding sites. These include 4145 cm^−1^; the aromatic combination CH_2_ of mAb, 4195 cm^−1^; OH stretch 1st overtone of glucose, 5102 cm^−1^; OH combination of sucrose/glucose, fructose (s/g/f), 5350 cm^−1^; RCO_2_H of mAb, 6108 cm^−1^; aromatic CH 1st overtone of mAb, 6568 cm^−1^; OH stretch of glucose, 6617 and 6729 cm^−1^; OH stretch of glucose. Clearly, significant amounts of OH first overtone bands of glucose are shown between 6000 and 7000 cm^−1^, respectively [51,55,56,57,59,60,61,62].

**Figure 11:**
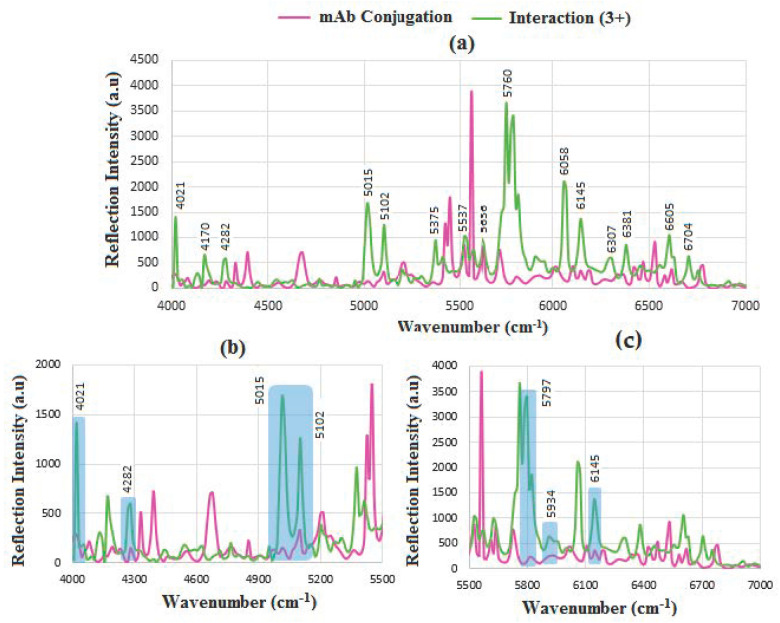
FT-NIR reflection signals for (**a**) before and after interaction of mAb with HER-II (3+), (**b**) the enlarged range between 4000 and 5400 cm^−1^, and (**c**) 5400–7000 cm^−1^ showing the areas of overlapped spectra corresponding to the presence of mAb and carbohydrate components.

The result of mAb interaction with HER-II (3+) is shown in Figure 11a. The overlapped areas in Figure 11b,c represent 4021 cm^−1^ of CH_2_ aromatic combination of mAb, 4282 cm^−1^; OH 1st overtone of glucose, 5015 cm^−1^; 2nd overtone/combination mods of mAb, 5102 cm^−1^; OH 1st stretch and CO stretch combination of (s/g/f) of carbohydrate, 5797cm^−1^; OH 1st overtone of glucose, 5934 cm^−1^; OH 1st overtone of glucose, 6145 cm^−1^ aromatic CH 1st overtone of mAb, respectively [44,48,49,50,52,53,54,55]. It seems that the most significant peaks in this case correspond to carbohydrate components, particularly glucose at the 1st OH stretch.

**Figure 12:**
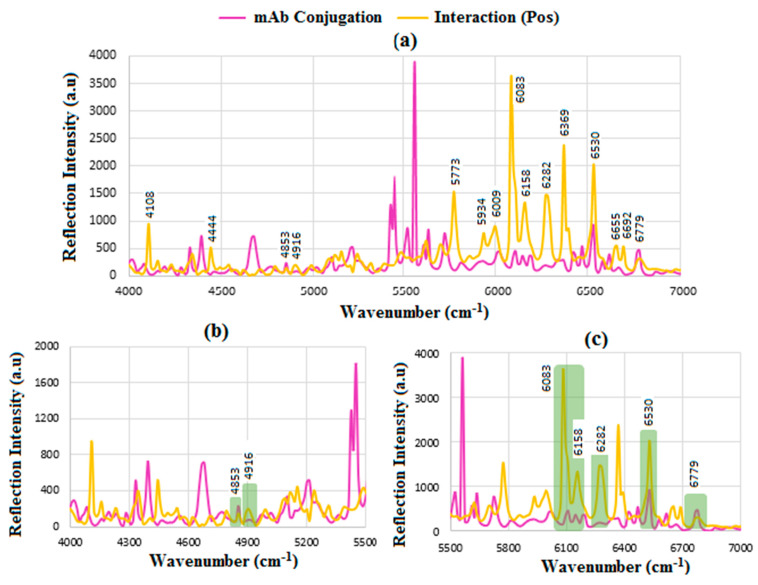
FT-NIR reflection signals for (**a**) before and after interaction of mAb with HER-II (Pos), (**b**) the enlarged range between 4000 and 5400 cm^−1^, and (**c**) 5400–7000 cm^−1^ showing the areas of overlapped spectra corresponding to the presence of mAb and carbohydrate components.

Similarly, Figure 12a shows the results for mAb interaction with HER-II positive where most significant peaks are related to OH 1st overtone of glucose, aromatic CH 1st stretch overtone, and CH combination/CH 1st overtone bands of polysaccharides. The major overlapped spectra in Figure 12b,c are 4853 cm^−1^ corresponding to CH combination/CH 1st overtone bans of polysaccharides, 4916 cm^−1^; CONH_2_ combination CN-H stretch, 6083 and 6158 cm^−1^; aromatic CH 1st stretch overtone, 6282 cm^−1^; CH combination/CH 1st overtone bans of polysaccharides, 6530 cm^−1^; OH 1st overtone of glucose and possibly RNH_2_ 1st overtone, and 6779 cm^−1^; RNH_2_ 1st overtones, respectively.

**Figure 13:**
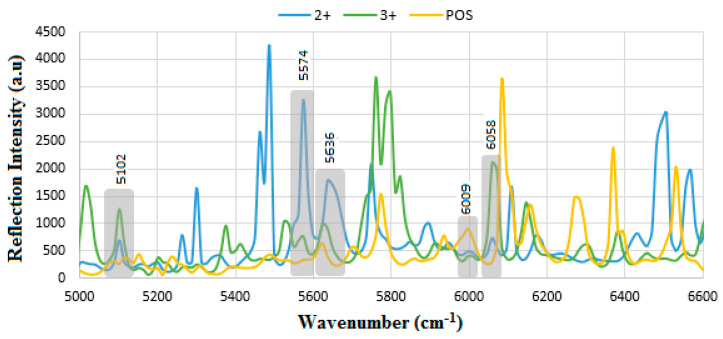
Comparison of the FT-NIR reflections for the HER-II biomarkers 2+, 3+, and Pos. 2+ shows the most abundant molecular bands followed by positive and 3+, respectively.

The comparison of the FT-NIR biomarkers is given in Figure 13, where the most dominant spectra belong to 2+, Pos, and then 3+ corresponding to OH-combination of s/g/f, RNH_2_ 1st overtone, and aromatic CH 1st overtone of mAb, respectively. The common biomolecular bands of the biomarkers are the overlapped areas that can be utilized as a common indicator for the analysis of the analyte.

### 6.6. SERS

**Figure 14:**
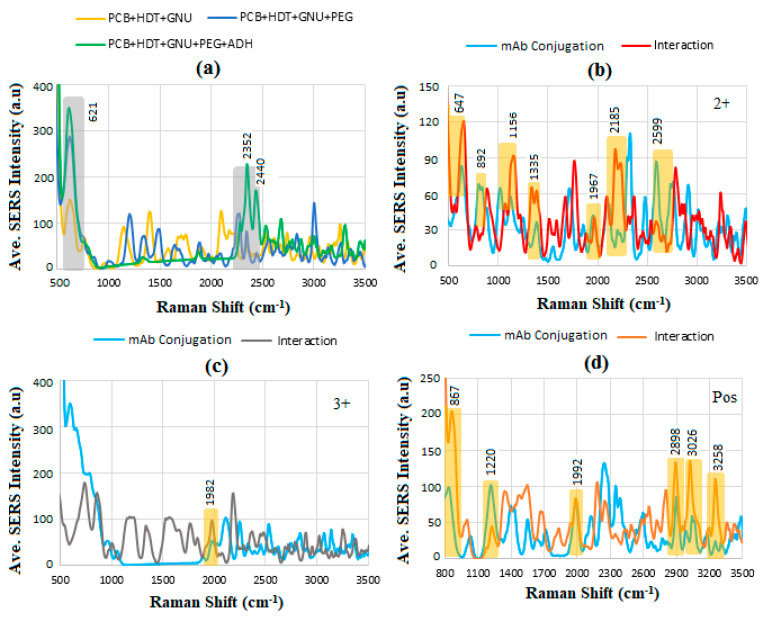
Variation in average SERS intensity with Raman shift for (**a**) PCB functionalization, before and after interaction of mAb with (**b**) 2^+^, (**c**) 3^+^, and (**d**) Pos, respectively.

Figure 14a shows the step-wise functionalization of PCB where the intensity of overlapped peaks at 621 corresponding to phenylalanine increases as the next layer is added. Other overlapped peaks at 2352 and 2440 cm^−1^ represent the NH_3_^+^ I amine present in ADH as a symmetrical molecule with a C4 backbone and the reactive group of C=ONHNH_2_. The most important feature of BC SERS diagnosis is finding the common peaks that overlap on the same wave number, indicating the Raman shift. Figure 14b shows the results for HER-II (2^+^) biomarker interaction with mAb, where clearly the intensity increases after the interaction due to the abundance of protein contents. However, the overlapped spectra can be utilized as an indicator corresponding to particular molecular bands. Using data from Table 4 [41,57,63,64], the 647 cm^−1^ band corresponds to tyrosine, 892 cm^−1^ to glycine, alanine ν (C-N-C) assigned to the symmetric C-N-C stretch mode, 1156 cm^−1^ to C-O benzoic ester and C-OH stretch alcohols, 1335 cm^−1^ to tryptophan and α helix, 1967 cm^−1^ to C-N amines, N-H primary and secondary amines and amides, NH_3_ amine, and O-H stretch, 2185 cm^−1^ to N=C stretch, and 2599 cm^−1^ to O-H stretching, respectively. In the case of HER-II (3^+^), shown in Figure 14c, a relatively improved response was achieved between 1000 and 2000 cm^−1^. Still, many significant peaks were overlapped except at 982 cm^−1^, which corresponds to C-N amines, N-H primary and secondary amines and amides, NH_3_ amine, and O-H stretch. For the sample HER-II (Pos) in Figure 14d, a number of peaks meaningfully coincided, including at 867 cm^−1^ pertaining to glycine, alanine ν (C-N-C) assigned to the symmetric C-N-C stretch mode, 1220 cm^−1^ to C-O stretching carboxylic acid, 1220 cm^−1^ to C-O stretching carboxylic acid, 1992 cm^−1^ to C-N amines, N-H primary and secondary amines and amides, NH_3_ amine, and O-H stretch, 2898 cm^−1^ to C-H antisymmetric and symmetric stretching, 3026 cm^−1^ to C-H stretch olefin, and 3258 cm^−1^ to N-H stretch primary amide, respectively.

**Figure 15:**
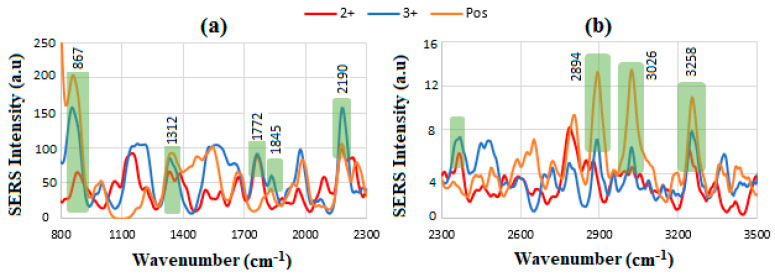
SERS results for HER-II 2^+^, 3+, and Pos, respectively, (**a**) between 800 and 2300 cm^−1^ and (**b**) between 2300 and 3500 cm^−1^, where the dominant overlapped peaks can be used as indicators for detection and comparison of the biomarkers.

The comparison of all HER-II biomarkers is shown in Figure 15, where the most identified dominant and common peaks exhibit an increasing intensity in order of 2^+^, 3^+^, and Pos, respectively. These peaks include 867 cm^−1^ corresponding to glycine, alanine *ν* (C-N-C) assigned to the symmetric C-N-C stretch mode, 1312 cm^−1^ to tryptophan and α helix, 1772 cm^−1^ being almost equal for 2^+^ and 3^+^ is possibly related to C=O antisymmetric stretch, 1845 cm^−1^ probably related to C=O stretch, 2190 cm^−1^ to C=N stretch, which is higher in the 2^+^ sample, 2894 cm^−1^ to C-H antisymmetric and symmetric stretching, 3026 cm^−1^ to NH_3_^+^ in amino acids, and 3258 cm^−1^ to N-H stretch primary amide, respectively. The results are justifiable because the BC samples correspond to early, locally advanced, and meta stages, respectively.

**Figure 16:**
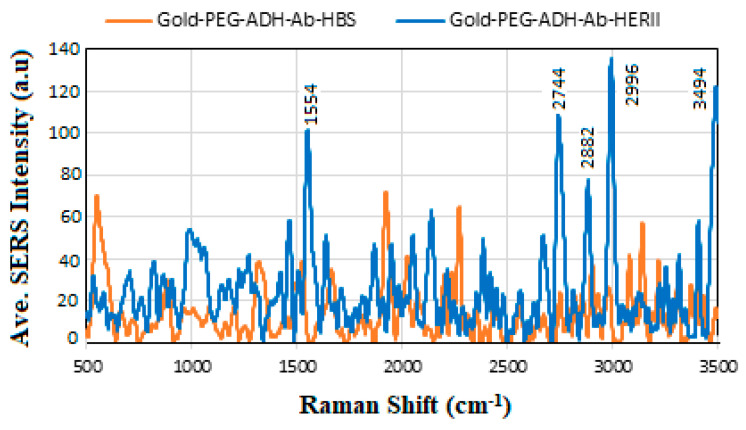
Comparison of SERS spectra for HBS and BCS.

The comparison of SERS spectra for HBS and BCS is shown in Figure 16, where the enhanced intensity of HER-II is due to the presence of additional proteins and carbohydrates associated with the cancerous serum. The most dominant lines include 1554 cm^−1^ corresponding to the COO- in carboxylic acid salts, 2744 cm^−1^ shows the O-H stretching, 2882 and 2996 cm^−1^ are likely related to the C-H antisymmetric and symmetric stretching, and 3494 cm^−1^ indicates the presence of the C-H antisymmetric, C-H stretch, NH_3_ in amino acids, N-H primary and secondary, respectively.

There are other peaks observed in the interaction results but not necessarily overlapped, which corresponds to the presence of some carbohydrates mainly, (a) monosaccharides such as glucose, fructose, and galactose, (b) disaccharides such as sucrose (glucose + fructose), lactose (glucose + galactose), and maltose (glucose + glucose), and (b) polysaccharides, which are the most abundant carbohydrates found in food. They are long-chain polymeric carbohydrates composed of monosaccharide units bound together by glycosidic linkages. The results showed some peaks, including 1398 cm^−1^ corresponding to D-(−)-fructose, 1481 cm^−1^ to D-(+)-galactose, 2657 cm^−1^ to 2-dexy-D-ribose, and 3393 cm^−1^ to D- (+)-glucose, respectively. Table 5 summarizes some of the carbohydrates that can be found in BCS.

### 6.7. Statistical Analysis

To explore distinctions among spectra files, Principal Component Analysis (PCA) and Linear Discriminant Analysis (LDA) were utilized to process and classify variables. PCA transforms a set of observations from correlated variables into linearly uncorrelated variables known as principal components. When paired with LDA, PCA allows for the creation of a linear combination of features that have been grouped or separated into multiple categories to build a predictive model. Both PCA and LDA are techniques for reducing dimensionality, condensing a complex dataset into a smaller set of dimensions that retain essential information while discarding less critical details.

In our study, we condensed Raman spectra containing 3000 intensity values corresponding to 3000 wavenumbers and, similarly, FT-NIR spectra with approximately 4000 intensity values corresponding to 4000 wavenumbers. These original intensity values were reduced to three dimensions known as the three principal components. These intensity values act as the predictors (independent variables), while the identity of the scanned sample serves as the response variable (dependent variable). Partial Least Squares Regression (PLS regression) was employed using *sklearn.cross_decomposition.PLSRegression*, a function provided by Scikit-Learn. Dissimilar to traditional PCA, which focuses solely on predictors or independent variables (X), PLS Regression determines principal components in the predictor dimensions to explain the relationship between X and the response or dependent variable (Y). In our case, this entails reducing the intensity values to a set of principal components that elucidate the relationship between intensity and the spectrum’s identity.

**Figure 17:**
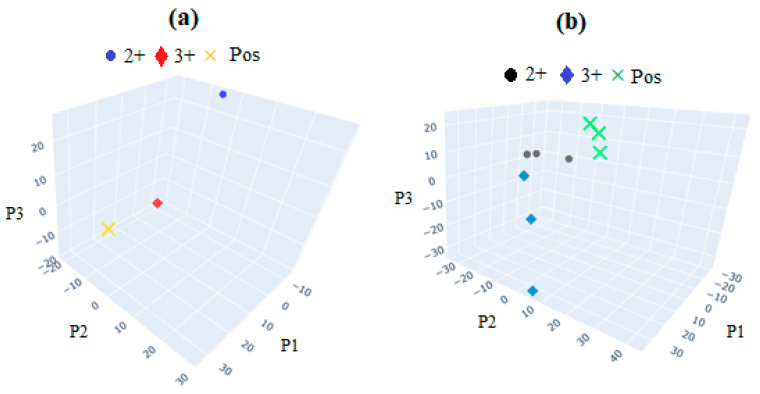
PCA plots of (**a**) FT-NIR and (**b**) SERS after interaction with HER-II in BCS. The groups within each sample are easily distinguished by PCA-LDA. The group members are closer to each other than in the case of FT-NIR, i.e., more statistically meaningful; however, both groups are clearly distinguishable.

Thus, the distinctions among these principal components will contain valuable information regarding the classification of spectra into respective sample groups. Ideally, all scans from a single sample group would cluster closely together on the PC plot, indicating high measurement precision (i.e., scans of the same sample are very similar to each other). In this ideal scenario, each sample group would also exhibit distinct separation from other groups, suggesting that the spectra from different groups are easily distinguishable and not similar in appearance. The interpretation of PCA-LDA results can be visualized and understood through PC plots. In all the plots presented in Figure 17, the spectra of GNU-conjugated mAb and mAb-HER-II are clearly separated into distinct groups. This separation enabled LDA to distinguish between the two groups with perfect accuracy, achieving a classification accuracy of 100% in all instances.

## 7. Conclusions

It was demonstrated that the fabricated SERS free-label immunosensor based on PANTF with EF of 0.3 × 10^5^ can detect the biomarkers in patients BCS with overexpressed HER-II of 2+, 3+, and Positive, respectively. Functionalization and conjugation were confirmed by UV-vis spectroscopy, and FT-NIR and SERS were used to identify the dominant molecular assignments before and after the mAb-HER-II interaction. The results indicated the presence of proteins and carbohydrate components, which may exist in BCS. Considering the type and stage of the samples, the SERS results for Pos were higher than 3+ and higher than 2+. PCA showed meaningful and distinguishable results, and LDA was able to differentiate the two groups with 100% accuracy (i.e., =1) in all cases. Much research is left to be conducted to advance the SERS immunosensor for practical clinical application, including improving the EF, testing different types of biomarkers, multiplexing, and automation of data reading and analysis. These are currently under investigation in our lab, and the results will be reported in the future.

## Figures and Tables

**Table 1:** Summary of BCS and mAb used in the experiment.

HER-II Type	Inventory Barcode	Cancer Stage and Group	mAb
2+	2399645	Early	MAB1129, R&D Systems
3+	201608071	IIIB-Locally advanced	HER-II CAU29848, Biomatik
POS	2572228	IIIB -Meta	HER-II CAU29848, Biomatik

**Table 2:** FT-NIR bands for tentative assignment of PEGylated GNU.

Band Assignment	Wavenumber (cm^−1^)	Wavelength (nm)
C-H combination	4082–4545	2449–2200
CH_2_	4140–4444	2415–2250
C-C	4444–4651	2250–2150
O-H combination	4545–5000	2200–2000
Combined C-H stretch and C-O	4690	2132
Combined C-H stretch and O-H	4890	2045
C=O stretch 2nd overtone	5150	1942
O-H 1st stretch overtone	5222–5333	1915
C-H 1st stretch overtone	5556–6061	1875–1650
CH_2_ 1st stretch overtone	5714–5988	1750–1670
O-H 1st stretch overtone	6000–7000	1666–1429
O-H 1st stretch overtone	6667–7143	1500–1400
CH_2_ combination 1st overtone	6944–7272	1440–1375
C-H combination overtone	7042–7692	1420–1300

**Table 3:** FT-NIR bands for tentative assignment of conjugated mAb-conjugated GNU.

Band Assignment	Wavenumber (cm^−1^)
Combination modes	4000–5000
CH_2_ aromatic combination	4000–4255
CH_2_ combination	4140–4444
CH_2_ aliphatic combination	4200–4500
CH_2_ 1st overtone	5714–5988
CH_2_ combination 1st overtone	6944–7272
β-sheet	4060
α-helix	4090 4365–4370
C-H combination	4098–4395
C-H 1st overtone	5633–5899
C-H combination 1st overtone	6849–7042
β-sheet	4405 4525–4540
RNH_2_ combination	4535–4683
RNH_2_ 1st overtone	6600–6734
α-helix	4615
CONH_2_(R) combination (N-H stretching)	4705–4926
β-sheet	4865
2nd Overtones and Combination modes	5000–8000
RCO_2_H 1st overtone (O-H stretching)	5249–5333
α-helix	5755
β-sheet	5915–5925
Aromatic CH 1st overtone	6060–6211
Aromatic OH 1st overtone	7017–7272

**Table 4:** The Raman shifts associated with mAb and proteins.

Tentative Assignment	Wavenumber (cm^−1^)
Tryptophan *ν*(C-S)	449
*ν*(C-S)	465
*ν*(S-S)	498
C-N-C in amines, Vib. modes of amino acids	510–400
*ν*(S-S)	512
S-S str. disulfide	550–450
Vib. modes of amide, C-I str. iodine derives	600–480
C=O in amides	615–535
Phenylalanine	622
Tyrosine	642, 640–650
Tryptophan, *δ*(-C-H)	722, 779
C-H out-of-plane bend phenyl, Vib. modes of acids	770–730
Glycine, alanine v (C-N-C) assigned to the symmetric C-N-C stretch mode	850–900
C-H, out-of-plane bend	980–965
Phenylalanine	1000–1010, 1103
Phenylalanine	1000–1010
C-N Amines	1000–1350
Str. vib. mode C-C in the phenyl ring	1000–1110
*ν*(C-C) or Alkyl C-N	1032–1170
C-O benzoic ester, C-OH str. alcohols	1100–1200
Phenylalanine	1103
S=O str. sulfuric ester	1150–1230
Tyrosine	1166
Tyrosine, *ν*(-C-N)	1175
Tyrosine, ν (-C-N)	1185–1175
C-O stretching carboxylic acid	1210–1320
Tryptophan	1220
β-sheet structure, amide III	1220–1240
Amide III	1230–1282
Amide III region δ (N-H, Cα-H), v (Cα-N)	1230–1340
C-O str., acetic ester	1232–1256
C-O str. benzoic ester	1250–1310
Tryptophan, α helix	1300–1345
Vib. mode in phospholipids	1392
C-O str. Carboxylic acid, Vib. mode in phospholipids,	1395–1400
Histidine	1400–1420
C-H scissor in CH_2_	1436–1458
N-H Primary and secondary amines and amides (stretch and bend)	1550–1640
COO^−^ in carboxylic acid salts	1560–1610
NH_2_ in primary amines	1580–1650
C-O asym. str. -COO^−^ carboxylate	1610–1540
Tryptophan *ν*(C=C), Tyrosine	1620
N-H in primary amides	1620–1650
C=O stretch in secondary amide (Amide I)	1630–1680
C=O stretch in primary amide (Amide I)	1650–1670
Amide I, α helix	1654
C=O stretch (fairly broad)	1690–1710
C=O vibration	1720–1740
C=O antisym stretch	1790–1870
C=O stretch	1800–1820
C-N amines, N-H primary and secondary amines and amides (stretch and bend), NH_3_ amine, and O-H stretch	1920–2300
N=C stretch	2115–2175
C=N stretch	2200–2260
N=C=O asym. str. isocyanate	2271
P-H stretch	2280–2410
NH_3_ I amine, and O-H stretch	2350–3100
-NH_3_^+^ I amine	2350–2750
O-H stretch (broad)	2400–3100
O-H stretching	2560–2750
C-H antisym and sym stretching	2850–2990
=C-H stretch in aromatic and unsaturated hydrocarbons	3000–3100
NH_3_^+^ in amino acids	3000–3200
C-H str. olefin	3010–3040
C-H str. olefin	3050–3155
C-H str. olefin	3075–3095
NH_3_ amine salt	3145–3355
C-H antisymmetric, C-H stretch, NH_3_ in amino acids, N-H primary and secondary	3100–3500
N-H str. primary amide	3250–3420
-NH_2_ in aromatic amines, primary amines and amides	3320–3520
-NH_2_ in primary amides	3340–3360
N-H stretching associated NH, amine	3500–3060

**Table 5:** SERS results for carbohydrate-related assignments of BCS.

SERS Shift (cm^−1^)	Assignment
304	D-(+)-galactose amylopectin
345	D-(+)-maltose
439	D-(+)-glucose
452	D-(+)-raffinose pentahydrate
699	L-(+)-arabinose
737	D-(+)-sucrose
770–730	D- (+)-sucrose
810–833	D-(−)-ribose
880	2-dexy-D-ribose
935	D-(+)-raffinose pentahydrate
950–810	D- (+)-raffinose pentahydrate
958,1369	D (+) mannose
1104	D-(+)-xylose D-(+)-galactose
1398	D-(−)-fructose
1481	D-(+)-galactose
2665	2-dexy-D-ribose
2865	2-dexy-D-ribose
2919	D-(−)-ribose
3019	D-(−)-fructose
3204	D-(+)-galactose
3393	D-(+)-glucose
3397	D-(+)-glucose

## Data Availability

All data generated or analyzed during this study are included in this published article.
